# Designing a Stable g-C_3_N_4_/BiVO_4_-Based Photoelectrochemical Aptasensor for Tetracycline Determination

**DOI:** 10.3390/toxics11010017

**Published:** 2022-12-25

**Authors:** Lu Qiao, Yue Zhao, Yuanyuan Zhang, Mingjuan Zhang, Yani Tao, Yao Xiao, Xinxia Zeng, Yi Zhang, Yuan Zhu

**Affiliations:** 1 College of Environmental Science and Engineering, Hunan University, Changsha 410082, China; 2 Key Laboratory of Environmental Biology and Pollution Control, Hunan University, Ministry of Education, Changsha 410082, China

**Keywords:** photoelectrochemical (PEC) aptasensor, tetracycline, efficient strategy, signal on, high stability

## Abstract

The excessive consumption of tetracycline (TC) could bring a series of unpredictable health and ecological risks. Therefore, it is crucial to develop convenient and effective detection technology for TC. Herein, a “signal on” photoelectrochemical (PEC) aptasensor was constructed for the stable detection of TC. Specifically, the g-C_3_N_4_/BiVO_4_ were used to promote the migration of photo-generated charges to an enhanced photocurrent response. TC aptamer probes were stably fixed on the g-C_3_N_4_/BiVO_4_/FTO electrode as a recognition element via covalent bonding interaction. In the presence of TC, the aptamer probes could directly recognize and capture TC. Subsequently, TC was oxidized by the photogenerated holes of g-C_3_N_4_/BiVO_4_, causing an enhanced photocurrent. The “signal on” PEC aptasensor displayed a distinguished detection performance toward TC in terms of a wide linear range from 0.1 to 500 nM with a low detection limit of 0.06 nM, and possessed high stability, great selectivity, and good application prospects.

## 1. Introduction

Nowadays, various hazardous small molecules (i.e., antibiotics, insecticides, environmental pollutants, pesticide residues, and hormones) have become significant factors threatening environmental health. Tetracycline (TC), a common hazardous small molecule, has been widely used as a feed additive in agriculture and livestock breeding on account of its economic advantage, broad-spectrum activity against bacteria, and favorable oral absorption [1,2]. However, the abuse of TC can cause residues in food-producing animals and environmental media, and the consumption of such food or exposure to contaminated soil/water can cause serious harm to the human body, such as allergic reactions, skeletal dysplasia, and hepatotoxicity [3,4]. Additionally, it can cause the spread of super bacteria with drug resistance and reduce the efficient treatment of bacterial infections [5]. Therefore, the development of efficient platforms for monitoring tetracycline or other hazardous small molecules is an objective and realistic demand. Currently, the main methods of TC detection are chromatographic detection, bioassay, and immunoassay, e.g., high-performance liquid chromatography (HPLC), fluorescence assay, lateral flow assay, and mass spectrometry [6,7,8,9,10]. Although these traditional techniques are able to detect TC, there are shortcomings, including high testing costs, complicated operations, long detection time, poor reproducibility, and so on. Thus, an efficient strategy for highly reliable TC detection is critical. 

Indeed, various sensors have been rising to the challenge in becoming a feasible technique to detect TC, on account of their simple operation, high selectivity, and low costs, such as biomimetic electrochemical sensor [11], electrochemical sensor [12], electrochemical immunosensor [13], fluorescent sensor [14], etc. Alternatively, the photoelectrochemical (PEC) aptasensor has been drawing broad attention, owing to its low background noise, great sensitivity, and fast response [15,16]. It is worth noting that the PEC aptasensor can also directly realize the expression of the response signal without extra signal indicators, which is so practical compared to other sensors [17,18,19]. In PEC construction, the selection of appropriate photoactive materials is favorable to obtain a high efficiency and stable signal output [20]. Many photoactive materials (e.g., inorganic semiconductors and organic materials) have been developed to construct the PEC aptasensor, due to their unique PEC properties [21]. Among them, graphitic carbon nitride (g-C_3_N_4_) has been widely explored owing to its feasible photoactivity, low cost, and appropriate bandgap [22]. Nevertheless, pure g-C_3_N_4_ shows poor PEC properties due to its quick recombination of photogenerated electrons/holes, which restricts the construction of a high-performance PEC aptasensor [23]. Therefore, it is vital to effectively address this issue. In fact, a number of methods, including element doping, morphological control, and heterojunction system have been reported [24,25]. Inspiringly, the g-C_3_N_4_-based heterojunctions as an uncomplicated method are able to significantly improve the PEC performance of g-C_3_N_4_ [26]. For instance, a proposed PEC sensor based on ZnIn_2_S_4_/g-C_3_N_4_ heterojunction was developed for the sensitive detection of bisphenol A [27]. BiVO_4_ as a magnetic metal oxide possesses considerable merits, i.e., high visible light response, low cost, and low toxicity [28]. Researchers have pointed out that BiVO_4_ was suitable for decorating g-C_3_N_4_ owing to their well-matched energy-level structure, and the designed heterojunction has promoted the fast transfer of electrons and exhibited attractive and stable photocurrent signal [29]. Thence, BiVO_4_/g-C_3_N_4_ heterojunction could be expected to improve an increased PEC performance and be applied to the design of a PEC aptasensor for detecting TC.

Herein, we fabricated a reliable PEC aptasensor based on g-C_3_N_4_/BiVO_4_ as an electrode material for TC determination. Additionally, the TC aptamer probes could be tightly assembled on the surface of g-C_3_N_4_/BiVO_4_ via covalent bonding interaction, to prevent it from falling off the electrode and further obtain a more stable and reliable signal response. When there is presence of TC, the aptamer probes could specially capture the TC and be oxidated by the accumulated holes on the photoactive materials and impede the electron–hole recombination, causing an enhanced photocurrent signal [30]. This work provided a promising method for a feasible analysis platform with excellent stability, selectivity, and detectability.

## 2. Experimental Section

### 2.1. Reagents and Instruments

Melamine (C_3_H_6_N_6_), sodium vanadate (Na_3_VO_4_·12H_2_O), bismuth nitrate (Bi(NO_3_)_3_·5H_2_O), chitosan powder (CS), glutaraldehyde (GA), bovine serum albumin (BSA), tetracycline (TC), oxytetracycline (OTC), kanamycin (KAN), tobramycin (TOB), and ciprofloxacin (CIP) were bought from Sinopharm Chemical Reagent Corporation. Additionally, the TC aptamer probes were supplied by Sangon Biotechnology Co.Ltd (sequences 5’ to 3’: NH_2_-(CH_2_)_6_-CGT ACG –GAA TTC GCT AGC CCC CCG GCA GGC CAC GGC TTG GGT TGG TCC CAC TGC GCG TGG ATC CGA GCT CCA CGT G) [31].

A scanning electron microscope (SEM, ZEISS Sigma 300, Oberkochen, Germany) was used to conduct morphology of the prepared materials. X-ray diffractometer (Panaco X’ Pert-PRO Bruker AXS D8 Advances, Almelo, Netherland) was employed for X-ray diffraction (XRD) analysis. X-ray photoelectron spectroscopy (XPS, Thermo Scientific K-Alpha, Waltham, America) was carried out to evaluate the chemical compositions of the material. LAMBDA 750 UV/Vis/NIR Spectrophotometer was run to evaluate the absorbance properties of the samples. Microinfrared spectroscopy (Nicolet iN10 MX Infrared Imaging Microscope) was performed to determine the structure of the materials. A CHI 660E workstation (CH Instruments Ins., Shanghai, China) was operated for PEC and electrochemical measurement.

### 2.2. Synthesis of g-C_3_N_4_

A ceramic crucible with 10 g melamine was placed in a muffle furnace and subsequently heated to 550 °C for 4 h. Secondly, the resulting product was further heated at 550 °C for 2 h to obtain pure g-C_3_N_4_ nanosheets [32].

### 2.3. Synthesis of g-C_3_N_4_/BiVO_4_

The g-C_3_N_4_/BiVO_4_ with different weight ratios were prepared by the same procedure [33]. Specifically, 5 g-C_3_N_4_ was dissolved in 100 mL water and agitated for 30 min. Then, Bi(NO_3_)_3_·5H_2_O and NH_4_VO_3_ with a molar ratio of 1:1 were dropped into the above solution, followed by stirring for 30 min. Then, the mixture was further rinsed and dried at 80 °C for 12 h and calcined to 400 °C for 2 h.

### 2.4. Fabrication of the PEC Aptasensor

First, 200 μL of 4 mg mL^−1^ g-C_3_N_4_/BiVO_4_ suspension was added to the fluorine-doped tin oxide (FTO) electrode to obtain the working electrode (g-C_3_N_4_/BiVO_4_/FTO electrode). Afterwards, 20 μL each of 0.1 wt% chitosan (CS), 5 wt% glutaraldehyde (GA), and 4 μM TC aptamer solution was added to the surface of the working electrode, so that the TC aptamer probes could be tightly immobilized to the surface of electrode via covalent bonding interaction. Finally, 20 μL of 1 wt% BSA was dropped on the above electrode to prevent non-specific adsorption [34,35].

### 2.5. Detection of TC 

Prior to the PEC detection, 20 μL of TC solution with different concentrations (0, 0.1, 1, 10, 50, 100, 500 nM) was dropped on the prepared electrode and incubated at room temperature for 60 min, followed by rinsing with 0.01 M PBS and testing PEC signal in Na_2_SO_4_ (0.1 M) solution [15,32].

## 3. Results and Discussion

### 3.1. Working Principle of the PEC Aptasensor

**Scheme 1:**
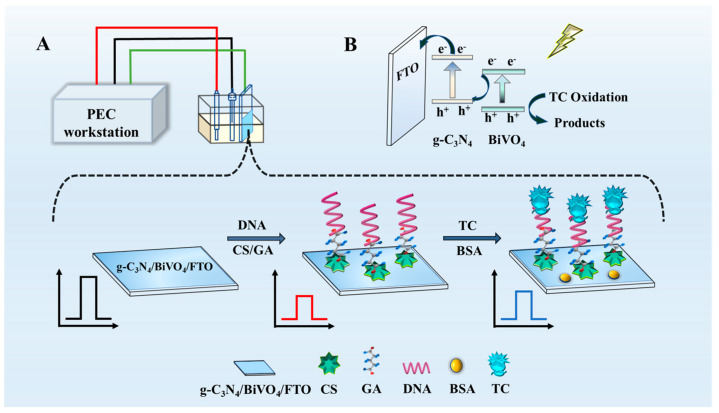
Schematic diagram of the development of PEC aptasensor for TC determination: (**A**) the assembly of the PEC aptasensor; (**B**) the mechanism of the PEC aptasensor.

Scheme 1 depicted the working principle of the proposed PEC aptasensor. In this progress (Scheme 1A), an FTO electrode was sequentially decorated by the g-C_3_N_4_/BiVO_4_, CS, GA, TC aptamer probes, BSA, and TC. Here, the g-C_3_N_4_/BiVO_4_ heterojunction was designed successfully owing to the well-matched energy level of g-C_3_N_4_ and BiVO_4_ (Scheme 1B), which exhibited enhanced separation of photogenerated carriers and an improved, attractive, and stable photocurrent signal, indicating that it is feasible to fabricate more an accurate PEC aptasensor [29,36]. Moreover, the aptamer probes were tightly immobilized onto the surface of g-C_3_N_4_/BiVO_4_/FTO, due to the covalent bonding interaction (GA was connected to both the amino-rich CS and the amino-functionalized TC aptamer probes). Remarkably, π-π stacking and covalent bond interaction are common methods to fix aptamer probes onto the sensor surface. The π-π stacking have merits of simple operation, low cost, and short reaction time. However, to a certain degree, the covalent bond interaction appears to be more stable, because this method could make the aptamer probes tightly bonded to the electrode and not easily fall off, so that the prepared aptamer sensor is able to present relatively stable results [5,15]. Notably, after the decoration of the TC aptamer probes, the photocurrent was decreased due to the steric hindrance of biomolecules. Then, the BSA was dropped onto the modified electrode to prevent non-specific adsorption. When there is existence of TC, the formation of aptamer–TC complexes brought about an increased photocurrent due to the oxidation by the holes accumulated on the g-C_3_N_4_/BiVO_4_, and further facilitated electrons transfer [30]. Specifically, the TC molecules could be oxidized directly by photogenerated holes in the valence band (VB) of BiVO_4_. Subsequently, the holes were consumed, which inhibited the recombination of photogenerated electrons/holes, resulting in the accumulation of more free electrons on the CB of g-C_3_N_4_, which were transferred to the electrode to generate an increased photocurrent signal (Scheme 1B). Therefore, a “signal on” PEC aptasensor for highly stable determination of TC was constructed.

### 3.2. Characterization of the Prepared Materials

**Figure 1:**
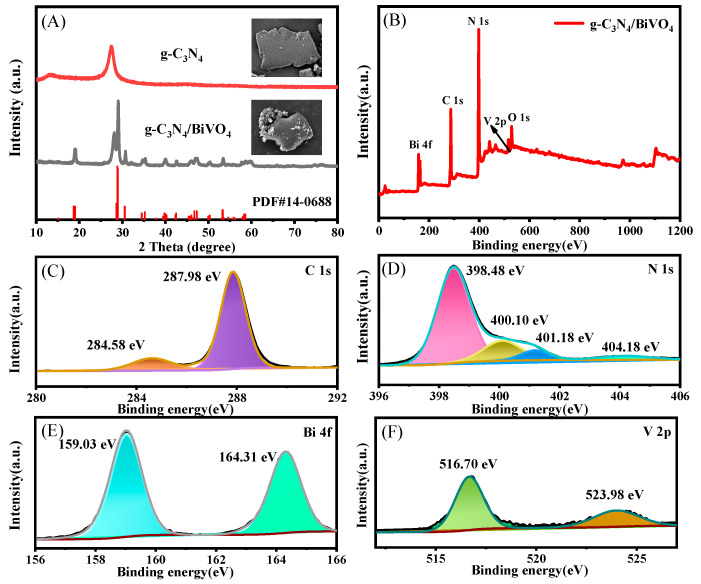
(**A**) SEM images and XRD patterns; (**B**) XPS survey spectrum; (**C**–**F**) corresponding high -resolution XPS spectra of the prepared materials.

The morphologies of the synthesized g-C_3_N_4_/BiVO_4_ were observed by SEM. As seen from Figure 1A, the g-C_3_N_4_ showed the appearance of smooth sheet structure. After the introduction of BiVO_4_, the irregular flower-like BiVO_4_ was directly anchored onto the surface of g-C_3_N_4_, confirming that the smooth surface of g-C_3_N_4_ could provide a suitable platform for the decoration BiVO_4_, and further indicating that the electrode material was successfully prepared. Figure 1A also exhibited the XRD patterns of g-C_3_N_4_ and g-C_3_N_4_/BiVO_4_ to characterize the crystalline features. As for g-C_3_N_4_, two main peaks around 13.1° and 27.3° were observed, which could be attributed to the (100) in-plane structural packing of heptazine units and (002) interlayer stacking of the conjugated aromatic segment [29]. After the BiVO_4_ decoration, the peaks of g-C_3_N_4_/BiVO_4_ were matched with the XRD standard card of BiVO_4_ (PDF#14-0688). Additionally, the diffraction peaks at 18.7°, 28.8°, 30.4°, 34.6°, 35.0°, 39.7°, 42.5°, 45.8°, 46.5°, 47.0°, 50.2°, and 53.1° are ascribed to the (1 1 0), (1 2 1), (0 4 0), (2 0 0), (0 0 2), (2 1 1), (1 5 0), (1 3 2), (0 4 2), (2 0 2), (1 6 1), and (1 5 2) diffraction phases of BiVO_4_, respectively [29]. Additionally, the peaks of g-C_3_N_4_ in the g-C_3_N_4_/BiVO_4_ were still retained but noticeably weakened owing to its low XRD intensity, and the existence of g-C_3_N_4_ in the composite could be verified by other characterization. 

XPS was employed to investigate the existence of g-C_3_N_4_ and the interface interaction between g-C_3_N_4_ and BiVO_4_ in the g-C_3_N_4_/BiVO_4_ composites. Figure 1B presented the full-scan XPS spectrum of the composites, showing C, N, Bi, V, and O elements were major compositions in g-C_3_N_4_/ BiVO_4_. In Figure 1C, the spectrum C 1s displayed two peaks located at 287.98 eV and 284.58 eV, which were attributed to the tertiary carbon C-(N)_3_ and the C-C bonds [37]. Four characteristic peaks at 398.48 eV, 400.10 eV, 401.18 eV, and 404.18 eV in the N 1s (Figure 1D) might be due to the sp^2^ hybrid nitrogen C=N-C, tertiary nitrogen N-(C)_3_, the nitrogen in the amino functional group C-N-H, and the π-excitation of C-N heterocycles, respectively [38]. The XPS analysis of C 1s and N 1s demonstrated that a triazine heterocyclic ring exists in the g-C_3_N_4_/BiVO_4_ composite. The peaks of Bi 4f (Figure 1E) at 159.03 eV and 164.31 eV were assigned to Bi 4f_7/2_ and Bi 4f_5/2_, respectively [29]. The results indicated that Bi^3+^ exists in the g-C_3_N_4_/BiVO_4_ composite. Additionally, in Figure 1F, the V 2p at 516.70 eV and 523.98 eV could be associated with V 2p_3/2_ and V 2p_1/2_, respectively [39]. Overall, these results illustrated that the g-C_3_N_4_/ BiVO_4_ was successfully prepared.

UV/vis diffuse reflectance spectrum (DRS) was performed to determine the optical absorption behaviors of the photoactive materials. In Figure S1A, the absorption edge of g-C_3_N_4_ was about 464 nm. After introducing BiVO_4_, the absorption edge of the complex was enhanced on account of the formation of a heterojunction between these two semiconductors [40]. Obviously, the g-C_3_N_4_/ BiVO_4_ could embrace more visible light which is of benefit to the PEC performance. Furthermore, Miro-FTIR spectra was adopted to confirm the chemical structures of the materials. As shown in Figure S1B, the g-C_3_N_4_/BiVO_4_ composites had similar absorption peaks of g-C_3_N_4_, suggesting no obvious structural change of g-C_3_N_4_ and g-C_3_N_4_/BiVO_4_ [38]. In conclusion, the g-C_3_N_4_/BiVO_4_ was successfully prepared, which could boost more charge transfer and will be helpful for the construction of the PEC sensor.

### 3.3. Fabrication of the PEC Aptasensor

**Figure 2:**
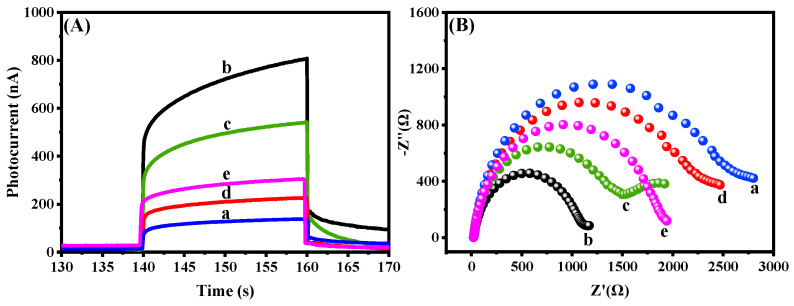
(**A**) Photocurrent responses and (**B**) electrochemical impedance spectra of (a) g-C_3_N_4_/FTO; (b) g-C_3_N_4_/BiVO_4_/FTO; (c) g-C_3_N_4_/BiVO_4_/Aptamer/FTO; (d) g-C_3_N_4_/BiVO_4_/Aptamer/BSA/FTO; (e) g-C_3_N_4_/BiVO_4_/Aptamer/BSA/TC/FTO.

To confirm the successful fabrication process of the PEC aptasensor, photocurrent measurement was conducted. In Figure 2A, the g-C_3_N_4_/BiVO_4_/FTO (curve b) displayed maximum photocurrent compared to the g-C_3_N_4_/FTO (curve a), owing to the appealing PEC properties of g-C_3_N_4_/BiVO_4_. However, after introducing aptamer probes and BSA, the photocurrent was certainly decreased due to their nonconductivity (curve c and curve d). Upon introduction of TC (curve e), the PEC response increased, which was attributed to the oxidation of TC by the accumulated holes on the prepared materials [41]. Electrochemical impedance spectroscopy (EIS) analysis can also be used to explain the feasibility of the PEC sensor. As illustrated in Figure 2B, g-C_3_N_4_/BiVO_4_/FTO electrode (curve b) presented a much smaller R_ct_ compared to g-C_3_N_4_/FTO (curve a), suggesting that BiVO_4_ enhanced the transfer of the electrons. After the aptamer probes and BSA were modified on the electrode (curve c and curve d), the R_ct_ increased gradually on account of the increased steric hindrance of the electrode interface [30]. During the presence of TC (curve e), the semicircles further increased, which was similar to the photocurrent results. These results confirmed successful modification of the PEC aptasensor.

### 3.4. Analytical Performance of the PEC Aptasensor

**Figure 3:**
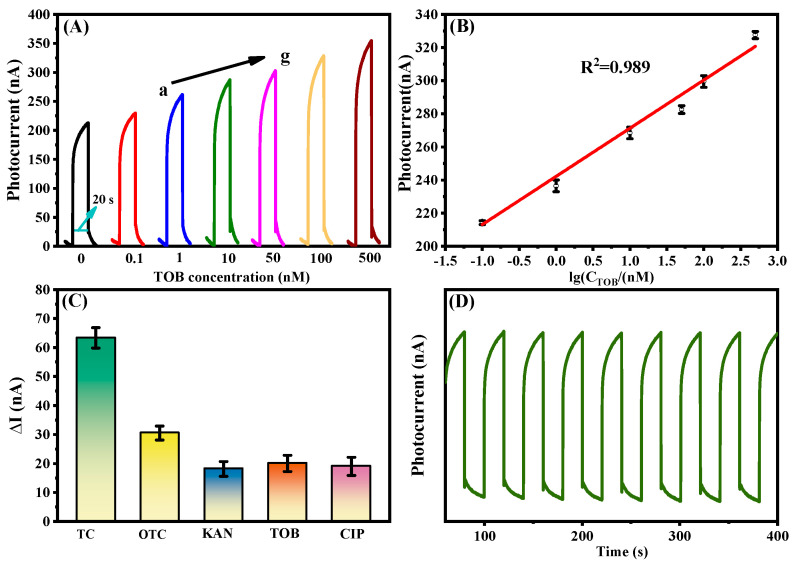
(**A**) Photocurrents of the PEC sensor at various concentrations of TC; (**B**) corresponding calibration curve; (**C**) selectivity; (**D**) stability of the PEC sensor.

To confirm the detection capacity of the PEC aptasensor, various concentrations (0, 0.1, 1, 10, 50, 100, 500 nM) of TC were studied under the optimal conditions (the specific optimization results were detailed in Figure S2). As shown in Figure 3A, the photocurrent intensity of the PEC aptasensor was enhanced with the concentration of TC increasing. Additionally, the linear equation could be fitted to I (nA) = 29.0622 lg (C/nM) + 242.209 (R^2^ = 0.989) in the range of 0.1-500 nM with a limit of detection (LOD) of 0.06 nM (S/N = 3) [42], which was below the maximum residue limits (MRLs) of TC (900 nM) in milk set by the US Food and Drug Administration [43]. Additionally, compared with most other methods reported previously, such as colorimetric, fluorescence, electrochemistry, and electrochemiluminescence (Table 1), the PEC aptasensor displayed excellent performance with a relatively lower detection limit and convenient operation.

Various interfering substances, such as OTC, KAN, TOB, and CIP, were chosen to evaluate the selectivity of the PEC aptasensor. As shown in Figure 3C, the photocurrent differences (∆I) of TC (10 nM) were different to the photocurrent differences of the four other interferents (10 nM), reflecting a high TC selectivity of the PEC sensor. Furthermore, the PEC aptasensor showed a wonderful stability under the eight on–off irradiation cycles (Figure 3D). Additionally, the long-term stability of the designed PEC aptasensor was determined. The results showed that when the PEC aptasensor was kept at 4 °C for 16 days, the current of the prepared electrode had no obvious change (Figure S3). These results demonstrate that the PEC aptasensor has favorable potential for TC detection.

### 3.5. Real Samples Analysis

A standard addition method was used to confirm the analytical feasibility of the PEC aptasensor in real samples. Table 2 exhibited that the recoveries of the PEC aptasensor ranged from 98.23–98.92%, and the RSD was 0.54–1.48%, which was essentially consistent with the results obtained by HPLC. Therefore, the fabricated PEC aptasensor has excellent application prospects.

## 4. Conclusions

In this work, we successfully fabricated a “signal on” PEC aptasensor based on the g-C_3_N_4_/BiVO_4_ for the highly stable determination of TC. The g-C_3_N_4_/BiVO_4_ acted as a photoactive material, which provided a stable and strong photocurrent. Moreover, TC aptamer probes were stably assembled on the surface of g-C_3_N_4_/BiVO_4_ via covalent bonding interaction to obtain a more reliable signal response. Due to the oxidation of the aptamer–TC complex by the accumulated holes on the g-C_3_N_4_/BiVO_4_, the “signal on” PEC aptasensor displayed satisfactory stability and acceptable satisfactory detectability. Under the optimal conditions, the PEC aptasensor for TC detection can manifest a linear range of TC from 0.1 to 500 nM with a detection limit of 0.06 nM. Obviously, the proposed sensor presents a trend of accurate detection capability and early warning function and has the potential to control the environmental and health risks of such pollutants. Additionally, we hope to promote this detection method in the future with actual samples.

## Figures and Tables

**Table 1:** Various methods for TC determination.

No.	Method	Linear range	Detection limit	Reference
1	Colorimetric	0.011–1 μM	3.1 nM	[44]
2	Fluorescence	2.5–50 μM	19.0 nM	[45]
3	Electrochemistry	0.5–5 μM	18 nM	[46]
4	Electrochemiluminescence	0.011–22.2 μM	4.0 nM	[47]
5	PEC aptasensor	5–200 nM	1.6 nM	[5]
6	PEC aptasensor	0.1–500 nM	0.06 nM	This work

**Table 2:** Determination of TC in Real Samples Using the Developed Sensor.

Samples	Added (nM)	Found (mean^a^ ± SD^b^) (nM)		Recovery (%)	RSD (%)
		PEC Aptasensor	HPLC		
River water	0	0	0	-	-
	40	39.29 ± 0.58	38.74 ± 0.80	98.23	1.48
	80	79.04 ± 0.71	78.38 ± 0.95	98.80	0.90
	200	197.85 ± 1.08	192.21 ± 0.64	98.92	0.54

mean^a^, the average value of three measurements; SD^b^, standard deviation.

## Data Availability

The data presented in this study are available on request from the corresponding author. The data are not publicly available due to privacy reasons.

## Supplementary Materials

The following supporting information can be downloaded at: https://www.mdpi.com/article/10.3390/toxics11010017/s1, Real samples preparation; Validation by HPLC; Figure S1: (A) UV/vis diffuse reflectance spectrum and (B) Miro-FTIR spectra of the prepared materials; Figure S2: The effect of PEC performance on (A) Proportion of composites, (B) Incubation concentration of aptamer probes; Figure S3: The long-term stability of the developed PEC aptasensor.

## References

[1] Jia P., Bu T., Sun X., Liu Y., Liu J., Wang Q., Shui Y., Guo S., Wang L. (2019). A sensitive and selective approach for detection of tetracyclines using fluorescent molybdenum disulfide nanoplates. Food Chem..

[2] Wang X., Li L., Jiang H., Zhangsun H., Wang Q., Sun X., Wang L. (2022). Highly selective and sensitive fluorescence detection of tetracyclines based on novel tungsten oxide quantum dots. Food Chem..

[3] Hamilton L.A., Guarascio A.J. (2019). Tetracycline Allergy. Pharmacy.

[4] Wei C., Liu Y., Jiang A., Wu B. (2022). A pharmacovigilance study of the association between tetracyclines and hepatotoxicity based on Food and Drug Administration adverse event reporting system data. Int. J. Clin. Pharm..

[5] Zhao Z., Wu Z., Lin X., Han F., Liang Z., Huang L., Dai M., Han D., Han L., Niu L. (2023). A label-free PEC aptasensor platform based on g-C_3_N_4_/BiVO_4_ heterojunction for tetracycline detection in food analysis. Food Chem..

[6] Gil R.L., Amorim C.M.P.G., Montenegro M.d.C.B.S.M., Araújo A.N. (2022). Cucurbit[8]uril-Based Potentiometric Sensor Coupled to HPLC for Determination of Tetracycline Residues in Milk Samples. Chemosensors.

[7] Ahmed S.R., Kumar S., Ortega G.A., Srinivasan S., Rajabzadeh A.R. (2021). Target specific aptamer-induced self-assembly of fluorescent graphene quantum dots on palladium nanoparticles for sensitive detection of tetracycline in raw milk. Food Chem..

[8] Tian Y., Bu T., Zhang M., Sun X., Jia P., Wang Q., Liu Y., Bai F., Zhao S., Wang L. (2021). Metal-polydopamine framework based lateral flow assay for high sensitive detection of tetracycline in food samples. Food Chem..

[9] Jiang T., Peng Z., Xie M., Fang X., Hong Y., Huang Z., Gao W., Zhou Z., Li L., Zhu Z. (2020). Rapid analysis of tetracycline in honey by microwave plasma torch mass spectrometry with ablation samples. Anal. Methods.

[10] Kadam U.S., Hong J.C. (2022). Advances in aptameric biosensors designed to detect toxic contaminants from food, water, human fluids, and the environment. Trends Environ. Anal. Chem..

[11] Stoian I.A., Iacob B.C., Dudas C.L., Barbu-Tudoran L., Bogdan D., Marian I.O., Bodoki E., Oprean R. (2020). Biomimetic electrochemical sensor for the highly selective detection of azithromycin in biological samples. Biosens. Bioelectron..

[12] Ma X., Pang C., Li S., Xiong Y., Li J., Luo J., Yang Y. (2019). Synthesis of Zr-coordinated amide porphyrin-based two-dimensional covalent organic framework at liquid-liquid interface for electrochemical sensing of tetracycline. Biosens. Bioelectron..

[13] El Alami El Hassani N., Baraket A., Boudjaoui S., Taveira Tenorio Neto E., Bausells J., El Bari N., Bouchikhi B., Elaissari A., Errachid A., Zine N. (2019). Development and application of a novel electrochemical immunosensor for tetracycline screening in honey using a fully integrated electrochemical Bio-MEMS. Biosens. Bioelectron..

[14] Ma X., Du C., Zhang J., Shang M., Song W. (2019). A system composed of vanadium(IV) disulfide quantum dots and molybdenum(IV) disulfide nanosheets for use in an aptamer-based fluorometric tetracycline assay. Mikrochim. Acta.

[15] Qiao L., Zhu Y., Zeng T., Zhang Y., Zhang M., Song K., Yin N., Tao Y., Zhao Y., Zhang Y. (2022). Turn-off photoelectrochemical aptasensor based on g-C_3_N_4_/WC/WO_3_ composites for tobramycin detection. Food Chem..

[16] Xiao K., Zhu R., Du C., Zheng H., Zhang X., Chen J. (2022). Zinc-Air Battery-Assisted Self-Powered PEC Sensors for Sensitive Assay of PTP1B Activity Based on Perovskite Quantum Dots Encapsulated in Vinyl-Functionalized Covalent Organic Frameworks. Anal. Chem..

[17] Kadam U.S., Trinh K.H., Kumar V., Lee K.W., Cho Y., Can M.T., Lee H., Kim Y., Kim S., Kang J. (2022). Identification and structural analysis of novel malathion-specific DNA aptameric sensors designed for food testing. Biomaterials.

[18] Trinh K.H., Kadam U.S., Song J., Cho Y., Kang C.H., Lee K.O., Lim C.O., Chung W.S., Hong J.C. (2021). Novel DNA Aptameric Sensors to Detect the Toxic Insecticide Fenitrothion. Int. J. Mol. Sci..

[19] Zhang Y., Xiao J.Y., Zhu Y., Tian L.J., Wang W.K., Zhu T.T., Li W.W., Yu H.Q. (2020). Fluorescence Sensor Based on Biosynthetic CdSe/CdS Quantum Dots and Liposome Carrier Signal Amplification for Mercury Detection. Anal. Chem..

[20] Kong W., Qu F., Lu L. (2020). A photoelectrochemical aptasensor based on p-n heterojunction CdS-Cu_2_O nanorod arrays with enhanced photocurrent for the detection of prostate-specific antigen. Anal. Bioanal. Chem..

[21] Lu Y., Zhang B., Tian Y., Guo Q., Yang X., Nie G. (2020). An enhanced photoelectrochemical sensor for aflatoxin B1 detection based on organic-inorganic heterojunction nanomaterial: Poly(5-formylindole)/NiO. Mikrochim. Acta.

[22] Mao L., Xue X., Xu X., Wen W., Chen M.-M., Zhang X., Wang S. (2021). Heterostructured CuO-g-C_3_N_4_ nanocomposites as a highly efficient photocathode for photoelectrochemical aflatoxin B1 sensing. Sens. Actuators B Chem..

[23] Liu Y., Yan K., Zhang J. (2016). Graphitic Carbon Nitride Sensitized with CdS Quantum Dots for Visible-Light-Driven Photoelectrochemical Aptasensing of Tetracycline. ACS Appl. Mater. Interfaces.

[24] Hosseini-Hosseinabad S.M., Siavash Moakhar R., Soleimani F., Sadrnezhaad S.K., Masudy-Panah S., Katal R., Seza A., Ghane N., Ramakrishna S. (2020). One-pot microwave synthesis of hierarchical C-doped CuO dandelions/g-C_3_N_4_ nanocomposite with enhanced photostability for photoelectrochemical water splitting. Appl. Surf. Sci..

[25] Ye L., Wang D., Chen S. (2016). Fabrication and Enhanced Photoelectrochemical Performance of MoS(2)/S-Doped g-C(3)N(4) Heterojunction Film. ACS Appl. Mater. Interfaces.

[26] Dang X., Song Z., Zhao H. (2020). Signal amplified photoelectrochemical assay based on Polypyrrole/g-C_3_N_4_/WO_3_ inverse opal photonic crystals triple heterojunction assembled through sandwich-type recognition model. Sens. Actuators B Chem..

[27] Chen Q., Yuan C., Zhai C. (2022). Label-free photoelectrochemical sensor based on 2D/2D ZnIn_2_S_4_/g-C_3_N_4_ heterojunction for the efficient and sensitive detection of bisphenol A. Chin. Chem. Lett..

[28] Xu Y., Wen Z., Wang T., Zhang M., Ding C., Guo Y., Jiang D., Wang K. (2020). Ternary Z-scheme heterojunction of Bi SPR-promoted BiVO_4_/g-C_3_N_4_ with effectively boosted photoelectrochemical activity for constructing oxytetracycline aptasensor. Biosens. Bioelectron..

[29] Xu X., Zhang J., Tao F., Dong Y., Wang L., Hong T. (2022). Facile construction of Z-scheme g-C_3_N_4_/BiVO_4_ heterojunctions for boosting visible-light photocatalytic activity. Mater. Sci. Eng. B.

[30] Tang L., Ouyang X., Peng B., Zeng G., Zhu Y., Yu J., Feng C., Fang S., Zhu X., Tan J. (2019). Highly sensitive detection of microcystin-LR under visible light using a self-powered photoelectrochemical aptasensor based on a CoO/Au/g-C_3_N_4_ Z-scheme heterojunction. Nanoscale.

[31] Feng Y., Yan T., Wu T., Zhang N., Yang Q., Sun M., Yan L., Du B., Wei Q. (2019). A label-free photoelectrochemical aptasensing platform base on plasmon Au coupling with MOF-derived In_2_O_3_@g-C_3_N_4_ nanoarchitectures for tetracycline detection. Sens. Actuators B Chem..

[32] Peng B., Lu Y., Luo J., Zhang Z., Zhu X., Tang L., Wang L., Deng Y., Ouyang X., Tan J. (2021). Visible light-activated self-powered photoelectrochemical aptasensor for ultrasensitive chloramphenicol detection based on DFT-proved Z-scheme Ag_2_CrO_4_/g-C_3_N_4_/graphene oxide. J. Hazard. Mater..

[33] Xie Q., He W., Liu S., Li C., Zhang J., Wong P.K. (2020). Bifunctional S-scheme g-C_3_N_4_/Bi/BiVO_4_ hybrid photocatalysts toward artificial carbon cycling. Chin. J. Catal..

[34] Zhu X., Gao L., Tang L., Peng B., Huang H., Wang J., Yu J., Ouyang X., Tan J. (2019). Ultrathin PtNi nanozyme based self-powered photoelectrochemical aptasensor for ultrasensitive chloramphenicol detection. Biosens. Bioelectron..

[35] Zhang Z., Zhang M., Xu Y., Wen Z., Ding C., Guo Y., Hao N., Wang K. (2020). Bi3+ engineered black anatase titania coupled with graphene for effective tobramycin photoelectrochemical detection. Sens. Actuators B Chem..

[36] Wang Q., Lin Y., Li P., Ma M., Maheskumar V., Jiang Z., Zhang R. (2021). An efficient Z-scheme (Cr, B) codoped g-C_3_N_4_/BiVO_4_ photocatalyst for water splitting: A hybrid DFT study. Int. J. Hydrog. Energy.

[37] Wang Y., Tan G., Liu T., Su Y., Ren H., Zhang X., Xia A., Lv L., Liu Y. (2018). Photocatalytic properties of the g-C_3_N_4_/{010} facets BiVO_4_ interface Z-Scheme photocatalysts induced by BiVO_4_ surface heterojunction. Appl. Catal. B Environ..

[38] Deng Y., Tang L., Feng C., Zeng G., Wang J., Zhou Y., Liu Y., Peng B., Feng H. (2018). Construction of plasmonic Ag modified phosphorous-doped ultrathin g-C_3_N_4_ nanosheets/BiVO_4_ photocatalyst with enhanced visible-near-infrared response ability for ciprofloxacin degradation. J. Hazard. Mater..

[39] Chen F., Yang Q., Wang Y., Zhao J., Wang D., Li X., Guo Z., Wang H., Deng Y., Niu C. (2017). Novel ternary heterojunction photcocatalyst of Ag nanoparticles and g-C_3_N_4_ nanosheets co-modified BiVO_4_ for wider spectrum visible-light photocatalytic degradation of refractory pollutant. Appl. Catal. B Environ..

[40] Lu M., Li Q., Zhang C., Fan X., Li L., Dong Y., Chen G., Shi H. (2020). Remarkable photocatalytic activity enhancement of CO_2_ conversion over 2D/2D g-C_3_N_4_/BiVO_4_ Z-scheme heterojunction promoted by efficient interfacial charge transfer. Carbon.

[41] Okoth O.K., Yan K., Feng J., Zhang J. (2018). Label-free photoelectrochemical aptasensing of diclofenac based on gold nanoparticles and graphene-doped CdS. Sens. Actuators B Chem..

[42] Cakiroglu B., Ozacar M. (2018). A self-powered photoelectrochemical glucose biosensor based on supercapacitor Co_3_O_4_-CNT hybrid on TiO_2_. Biosens. Bioelectron..

[43] Yao R., Li Z., Liu G., Fan C., Pu S. (2021). Luminol-Eu-based ratiometric fluorescence probe for highly selective and visual determination of tetracycline. Talanta.

[44] Tang Y., Huang X., Wang X., Wang C., Tao H., Wu Y. (2022). G-quadruplex DNAzyme as peroxidase mimetic in a colorimetric biosensor for ultrasensitive and selective detection of trace tetracyclines in foods. Food Chem..

[45] Yang K., Jia P., Hou J., Bu T., Sun X., Liu Y., Wang L. (2020). Innovative Dual-Emitting Ratiometric Fluorescence Sensor for Tetracyclines Detection Based on Boron Nitride Quantum Dots and Europium Ions. ACS Sustain. Chem. Eng..

[46] Besharati M., Hamedi J., Hosseinkhani S., Saber R. (2019). A novel electrochemical biosensor based on TetX2 monooxygenase immobilized on a nano-porous glassy carbon electrode for tetracycline residue detection. Bioelectrochemistry.

[47] Deng B., Xu Q., Lu H., Ye L., Wang Y. (2012). Pharmacokinetics and residues of tetracycline in crucian carp muscle using capillary electrophoresis on-line coupled with electrochemiluminescence detection. Food Chem..

