# Supplementary material for: Designing a Stable g-C3N4/BiVO4-Based Photoelectrochemical Aptasensor for Tetracycline Determination
Source: Toxics. 2022 Dec 25;11(1):17. doi: 10.3390/toxics11010017 (PMC9865260; doi:10.3390/toxics11010017)
Supplement: Supplementary file 1 [file toxics-11-00017-s001.zip › toxics-2082547-supplementary.pdf]

Supporting Information for

Designing a Stable g-C_3N_4/$BiVO_4$-Based Photoelectrochemical Aptasensor for Tetracycline Determination

Real samples preparation

The real samples (Xiangjiang River (Changsha, China)), were pretreated before analysis. Specifically, the river water sample was collected and filtered by a filter paper to remove suspended substances. Subsequently, the filtrate was further treated with 0.22 μm membrane to filter the microorganisms [1]. Finally, different concentrations tetracycline (TC) was spiked into these supernatants respectively by the standard addition method.

Validation by HPLC

The high-performance liquid chromatography (HPLC) measurements were performed on an Agilent 1260 Infinity II, USA with a C18 column (150 mm × 4.6 mm). The analytical column was maintained at 30 °C and eluted with a mixture of acetonitrile and 0.01M oxalic acid water at a total flow rate of 0.2 mL/min. The injection volume was 20 μL. To obtain the unknown concentrations of the corresponding substances in the actual samples, we used standard calibration curves of six samples of known concentrations.

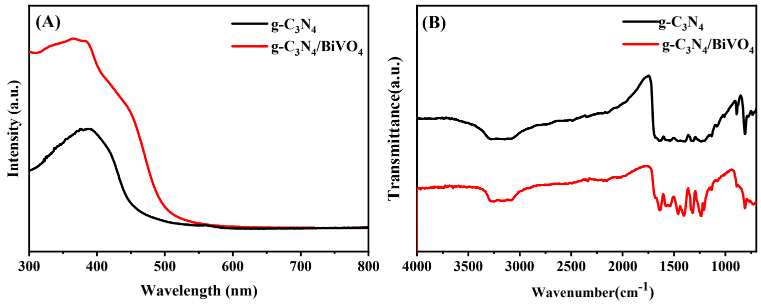

Figure S1. (A) UV/vis diffuse reflectance spectrum and (B) Miro-FTIR spectra of the prepared materials.

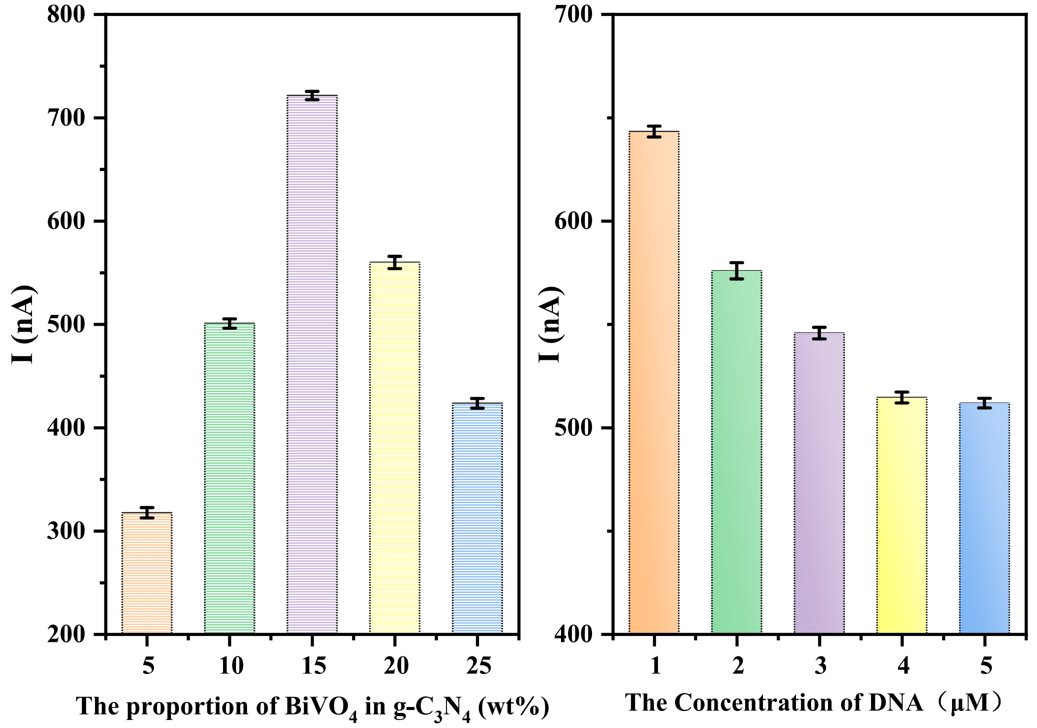

Figure S2. The effect of PEC performance on (A) Proportion of composites, (B) Incubation concentration of aptamer probes.

Given the initial photoelectric properties of this PEC sensor, different $BiVO_4$ content was mainly investigated. Fig. S2A shows that the maximum photocurrent appeared in the 15 wt% $BiVO_4$. Consequently, 15 wt% $BiVO_4$ becomes the optimal choice here. Furthermore, the aptamer probes concentration was investigated. As shown in Fig. S2B, with the increase of the concentration of the TC aptamer probes, the photocurrent decreased and achieved a balance at 4 μM of aptamer probes. Thus 4 μM of aptamer probes was the optimal choice [2].

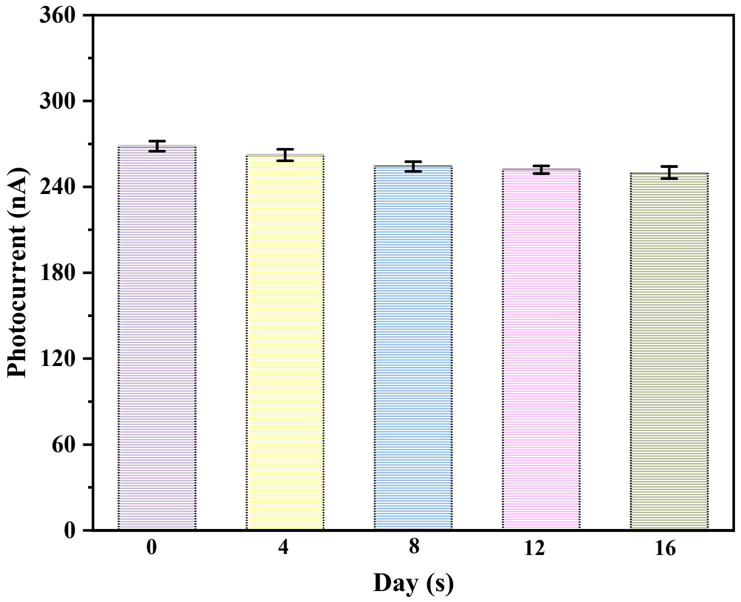

Figure S3. The long-term stability of the developed PEC aptasensor.

References

1. Zhu, X.; Gao, L.; Tang, L.; Peng, B.; Huang, H.; Wang, J.; Yu, J.; Ouyang, X.; Tan, J. Ultrathin PtNi nanozyme based self-powered photoelectrochemical aptasensor for ultrasensitive chloramphenicol detection. *Biosens Bioelectron* **2019**, *146*, 111756, doi:10.1016/j.bios.2019.111756.
2. Qiao, L.; Zhu, Y.; Zeng, T.; Zhang, Y.; Zhang, M.; Song, K.; Yin, N.; Tao, Y.; Zhao, Y.; Zhang, Y.; et al. "Turn-off" photoelectrochemical aptasensor based on g-C3N4/WC/WO3 composites for tobramycin detection. *Food Chem* **2022**, *403*, 134287, doi:10.1016/j.foodchem.2022.134287.
